# No significant differences in short-term renal prognosis between living kidney donors with and without diabetes

**DOI:** 10.1007/s10157-017-1487-5

**Published:** 2017-10-12

**Authors:** Takahiro Shinzato, Akira Kurosawa, Taro Kubo, Toshihiro Shimizu, Takaaki Kimura, Koji Nanmoku, Takashi Yagisawa

**Affiliations:** Department of Renal Surgery and Transplantation, Jichi Medical University Hospital, 3311-1 Yakushiji, Shimotsuke, Tochigi 3290498 Japan

**Keywords:** Living kidney donor, Diabetes, Renal function, Zero-time kidney biopsy

## Abstract

**Background:**

Renal prognosis in living kidney donors with diabetes is currently not known. In this study, we sought to investigate renal prognosis in living kidney donors with diabetes.

**Methods:**

We retrospectively investigated 241 living kidney donors who underwent nephrectomy at Jichi Medical University Hospital between January 2000 and December 2015. Donors with a follow-up period of less than 1 year were excluded. The remaining donors were divided into a diabetic group and a non-diabetic group. Their clinical parameters before donation and renal prognosis after donation were compared.

**Results:**

Of the 241 donors, 16 were excluded due to their follow-up period being less than 1 year. Of the remaining 225 donors, 14 were diabetic and 211 were non-diabetic. There were no significant differences in variables at pre-donation. The median follow-up period was 4.3 (1.5–10.7) and 4.6 (1.0–13.0) years in kidney donors with and without diabetes, respectively. At the end of follow-up, the estimated glomerular filtration rate was 51.7 ± 7.1 ml/min/1.73 m^2^ in the diabetic group and 52.1 ± 12.2 ml/min/1.73 m^2^ (*p* = 0.906) in the non-diabetic group; urine albumin excretion was 9.5 (2–251) mg/day (or mg/g creatinine) in the diabetic group and 6 (0–626) mg/day (or mg/g creatinine) in the non-diabetic group (*p* = 0.130); and urine protein excretion was 0.079 (0–0.41) g/day in the diabetic group and 0.051 (0–3.7) g/day in the non-diabetic group (*p* = 0.455).

**Conclusions:**

There were no significant differences in short-term renal prognosis between kidney donors with and without diabetes.

## Introduction

In a report at the Amsterdam Forum, it was determined that individuals with a history of diabetes or fasting blood glucose 126 mg/dl (7.0 nmol/l) on at least two occasions (or 2-h glucose with oral glucose tolerance test 200 mg/dl (11.1 mmol/l) should not donate [1]. Therefore, only a few cases of kidney transplantation from living kidney donors with diabetes may exist globally. On the other hand, in the Japanese criteria for living kidney donors, it is stated as follows: (1) donors should not be diabetic; (2) the fasting blood glucose level should be 126 mg/dl or less; and (3) the hemoglobin A1c (NGSP) level should be 6.2% or less, and it is desirable to perform 75 g oral glucose tolerance test if it is difficult to diagnose. Moreover, there are criteria for marginal kidney donors with diabetes, which require that donors should have: (1) a hemoglobin A1c (NGSP) level of at most 6.5%; (2) a urine albumin excretion of less than 30 mg/g creatinine (Cr); and (3) no history of insulin treatment. These criteria were published on 8 June 2014.

Meanwhile, there have been contradictory findings that diabetes is a risk factor for decline in renal function after nephrectomy for renal cell carcinoma or other illnesses [2–9]. Nevertheless, there have been no reports regarding the prognosis of kidney donors with diabetes. Therefore, the renal prognosis of kidney donors with diabetes is currently unknown.

In the present study, we investigated the renal prognosis of kidney donors with diabetes.

## Materials and methods

### Study design

We retrospectively reviewed the medical records of 241 consecutive living kidney donors who underwent nephrectomy at Jichi Medical University Hospital between January 2000 and December 2015. The observation period was from the time of kidney donation to 31 December 2016. Donors who had a follow-up period of less than 1 year were excluded. We stratified the donors into a diabetic group or non-diabetic group. We then compared the clinical parameters between the groups prior to donation as well as the estimated glomerular filtration rate (eGFR) and urinary albumin and protein excretion at 1 year after donor nephrectomy and at the end of follow-up. In addition, we evaluated the histological findings of zero-time kidney biopsies from donors with diabetes. We conducted multivariate analysis to investigate those factors that are predictive of eGFR and albuminuria.

### Definition of diabetes, hypertension, dyslipidemia, and smoking

Diabetes was defined as meeting the diagnostic criteria of diabetes mellitus [10] or having a documented history of diabetes treated with medications or diet therapy. Donors were considered to have hypertension if they had (1) a previous diagnosis or have been treated with medication or diet therapy, (2) a systolic blood pressure of 140 mmHg or greater, or (3) a diastolic blood pressure of 90 mmHg or greater. Donors were considered to have dyslipidemia if they had (1) a previous diagnosis or have been treated with medication or diet therapy, (2) a fasting serum LDL cholesterol of 140 mg/dl or greater, (3) an HDL cholesterol of less than 40 mg/dl, or (4) a triglyceride level of 150 mg/dl or greater. A smoker was defined as one who currently smoked or who had ever smoked.

### Calculation of eGFR and assessment of urine albumin and protein excretion

The eGFR was calculated by the method of Matsuo et al. [11] using the following equation: eGFR = 194 × Cr (mg/dl)^−1.094^ × age (years)^−0.287^ (× 0.739 if female) (ml/min/1.73 m^2^). Urine albumin and protein excretion were assessed by 24-h urine albumin and protein excretion. If there were no data of urine albumin excretion at the end of the follow-up period, spot urine albumin-to-creatinine ratio (mg/g Cr) was used.

### Histological study

We used the standard techniques to prepare the tissues for light microscopy: formalin fixing, paraffin embedding, and cutting at 1 mm thickness. The tissues were stained with hematoxylin and eosin, periodic acid-Schiff, silver methenamine–Masson trichrome, and Elastica van Gieson. Electron microscopy and immunofluorescence were not performed.

The histological findings were assessed by light microscopy, with semi-quantitative scores for glomerular mesangial expansion (score 0 = none or mild, 1 = mesangial area < capillary lumen, 2 = mesangial area = capillary lumen, 3 = mesangial area > capillary lumen), glomerular hypertrophy [(diameter of glomeruli ≥ 250 μm) score 0 = absent, 1 = present], polar vasculosis (score 0 = absent, 1 = present), arteriosclerosis (score 0 = no intimal thickening, 1 = intimal thickening with intima/media < 1, 2 = intimal thickening with intima/media ≥ 1), and arteriolar hyalinosis (score 0 = no hyalinosis, 1 = partial hyalinosis in one or more arterioles, 2 = hyalinosis in about 50% of arterioles, 3 = hyalinosis in more than 50% of arterioles or partial hyalinosis involving all layers of arterioles). In global glomerular sclerosis, interstitial fibrosis and tubular atrophy were evaluated as a percentage.

### Statistical analysis

Values for statistical analysis were expressed as the mean ± SD or median (range), when appropriate. Categorical variables were analyzed using the Chi-square test. Continuous variables were analyzed by student *t* test or Mann–Whitney *U* test, when appropriate. Independent predictors of postoperative low eGFR (< 45 ml/min/1.73 m^2^) and high urine albumin excretion (≥ 30 mg/day) were identified by logistic regression analysis. All baseline variables associated with decline in renal function or development of albuminuria after kidney donation were analyzed using univariate analysis. These included age, sex, BMI, diabetes, hypertension, dyslipidemia, smoking, eGFR, and urine albumin excretion. Independent variables were included in the models if the *p* value was less than 0.2 in the univariate analysis. Variables included in the final model were determined by backward stepwise selection with an inclusion criterion of *p* less than 0.05. *p* values less than 0.05 were considered significant. Statistical analysis was performed using EZR [12] version 1.35.

## Results

### Baseline characteristics

Preoperative characteristics of kidney donors with and without diabetes are shown in Table 1. Of the 241 donors, 16 were excluded, because they had a follow-up period of less than 1 year. Of the remaining 225 donors, 14 were diabetic and 211 were non-diabetic. There were no significant differences in these variables. In diabetic group, there were no cases without data for each parameters, while, in the non-diabetic group, there were no data for smoking in two cases, urine albumin excretion in 38 cases and urine protein excretion in 24 cases.

**Table 1:** Characteristics of donors with and without diabetes prior to donation

	Diabetes (*N* = 14)	Non-diabetes (*N* = 211)	*p* value
BMI (kg/m^2^)	24.8 ± 1.6	23.8 ± 3.4	0.259
Age (years)	59.1 ± 9.5	57.5 ± 10.6	0.593
Sex (male), *n* (%)	7 (50)	80 (37.9)	0.404
Hypertension (%)	50	23.7	0.0505
Dyslipidemia (%)	50	28	0.125
Smoking (%)	50	48.3	1
Serum creatinine (mg/dl)	0.64 ± 0.14	0.66 ± 0.15	0.579
eGFR (ml/min/1.73 m^2^)	88.5 ± 14.7	83.9 ± 17.4	0.329
Urine albumin excretion (mg/day)	9.75 (0–32)	8.00 (0–78)	0.482
Urine protein excretion (g/day)	0 (0–0.09)	0 (0–0.21)	0.113
ARB or ACE-I (%)	21.4	10	0.177

Values of categorical variables are presented as percentages (%) whereas continuous variables are reported as mean ± SD or median with range

BMI, age, serum creatinine and eGFR were compared between the groups using Student* t* test

Percentage of cases with male, hypertension, dyslipidemia, smoking history or administration of ARB or ACE-I were compared between the groups using Chi square test

Urine albumin and protein excretion were compared between the groups using Mann–Whitney* U *test

*BMI* body mass index,* eGFR *estimated glomerular filtration rate,* ARB* angiotensin receptor blockers,* ACE-I* angiotensin converting enzyme inhibitors

### Parameters of kidney donors with diabetes

The clinical parameters of donors with diabetes are shown in Table 2. In the diabetic group, whether retinopathy was present was unknown in six cases and the remaining eight cases did not exhibit retinopathy. The duration of diabetes was unknown in eight donors. They were found to be diabetic at the time of the screening test for kidney donation, because they had not received a medical check-up before then. Of the remaining six donors, the longest duration of diabetes was 6 years, and the shortest was 6 months. Regarding their treatment of diabetes, eight donors were treated with diet therapy, five donors were treated with oral therapy, and one donor was treated with insulin. The median (range) hemoglobin A1c level at the time of kidney donation was 6.0 (5.1–6.9)%. There were two donors with diabetes with hemoglobin A1c levels higher than 6.5%, urine albumin excretion rate of more than 30 mg/day, or a history of treatment with insulin. These donors had donated prior to the publication of the Japanese marginal donor criteria.

**Table 2:** Parameters of diabetic donors

	Case													
	1	2	3	4	5	6	7	8	9	10	11	12	13	14
At pre-donation														
Age (years)	65.0	73.4	44.6	74.2	48.2	57.2	61.3	55.7	61.6	59.2	59.1	42.9	57.0	67.8
Sex	F	M	F	F	M	M	F	M	F	M	M	M	F	F
BMI (kg/m^2^)	22.7	25.6	26.6	25.6	24.8	21.7	25.5	25.3	24.7	25.6	23.7	28.1	23.1	24.4
Retinopathy				No	No	No	No	No		No		No		No
Duration of diabetes (years)			1				6			2	0.5	2	1	
Hemoglobin A1c (%)	5.7	6.3	6.0	6.4	5.6	6.1	6.4	5.7	5.9	6.6	5.9	5.9	5.1	6.9
Treatment for diabetes	Oral	Insulin	Diet	Oral	Diet	Diet	Oral	Diet	Diet	Oral	Diet	Diet	Oral	Diet
Hypertension (%)	Yes	No	Yes	Yes	No	No	No	No	No	Yes	Yes	No	Yes	Yes
Dyslipidemia (%)	No	No	No	Yes	No	Yes	Yes	Yes	No	Yes	No	Yes	No	Yes
Smoking (%)	No	Yes	No	Yes	Yes	Yes	No	Yes	No	Yes	Yes	No	No	No
Urine albumin excretion (mg/day)	8.5	20	32	5	8	4	11	13	3	17	11	0	8	14
Urine protein excretion (g/day)	0	0	0	0.062	0.054	0	0	0	0	0	0.087	0	0	0.062
Serum creatinine (mg/dl)	0.44	0.72	0.59	0.53	0.88	0.82	0.53	0.81	0.47	0.75	0.73	0.54	0.54	0.54
eGFR (ml/min/1.73 m^2^)	106.2	81.0	85.8	83.4	73.3	75.4	88.1	77.1	100.4	82.4	84.9	129.4	88.1	83.9
Follow-up period (years)	10.7	7.0	2.0	8.0	5.5	5.7	5.2	4.5	4.0	3.9	3.5	3.0	2.4	1.5
At the end of follow-up														
Urine albumin excretion (mg/day or mg/g creatinine)	12^a^		134		2	5	10	9	77	251	20	3	8	7
Urine protein excretion (g/day)	(−)^b^	(±)^b^	0.269	0.115	0	0	0	0.076	0.233	0.412	0.082	0	0	0.088
Serum creatinine (mg/dl)	0.64	1.28	0.81	0.85	1.4	1.18	0.91	1.09	0.79	1.06	1.25	1.13	0.97	0.83
eGFR (ml/min/1.73 m^2^)	67.5	42.1	59.9	48.3	42.8	49.3	47.7	54.5	55.9	55.4	46.4	56.6	45.9	52.1

*BMI* body mass index,* eGFR* estimated glomerular filtration rate

^a^ Spot urine albumin (mg/g creatinine), ^b^ dipstick urinalysis for protein

### Histological findings of zero-time kidney biopsy

In one donor with diabetes, it was impossible to evaluate the zero-time kidney biopsy specimen, because it was processed inappropriately. Histological findings of the zero-time kidney biopsies from the remaining 13 donors are shown in Table 3. There were no cases with nodular or exudative lesions.

**Table 3:** Histological findings of 0-time kidney biopsy from donors with diabetes

	Case												
	1	3	4	5	6	7	8	9	10	11	12	13	14
Glomerular mesangial expansion (0–3)	1	1	0	1	1	1	1	1	2	1	1	1	1
Glomerular hypertrophy (0 or 1)	1	1	0	0	0	1	0	1	1	1	0	0	0
Global sclerosis (%)	23.7	14.3	5.6	6.3	0	18.2	30.0	0	28.6	14.3	0	0	4.0
Polar vasculosis (0 or 1)	0	0	0	0	1	0	1	0	1	0	0	0	0
Arteriosclerosis (0–2)	0	0	1	1	0	0	0	0	2	0	0	0	0
Arteriolar hyalinosis (0–3)	1	0	0	0	1	1	1	0	1	2	0	0	1
Interstitial fibrosis and tubular atrophy (%)	5	5	5	5	0	20	30	5	10	10	5	5	5

### After donation

**Fig. 1:**
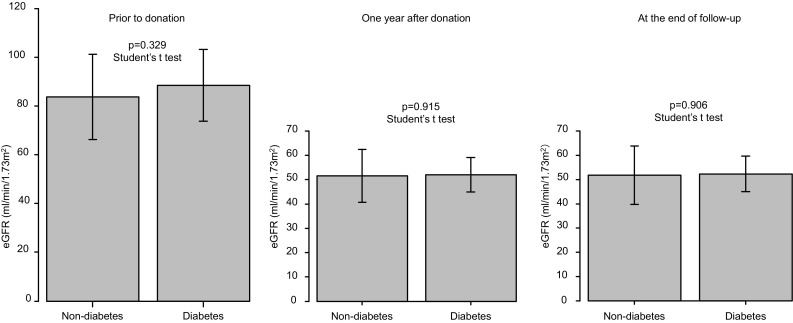
eGFR prior to donation, 1 year after donation, and at the end of follow-up*. eGFR* estimated glomerular filtration rate

**Fig. 2:**
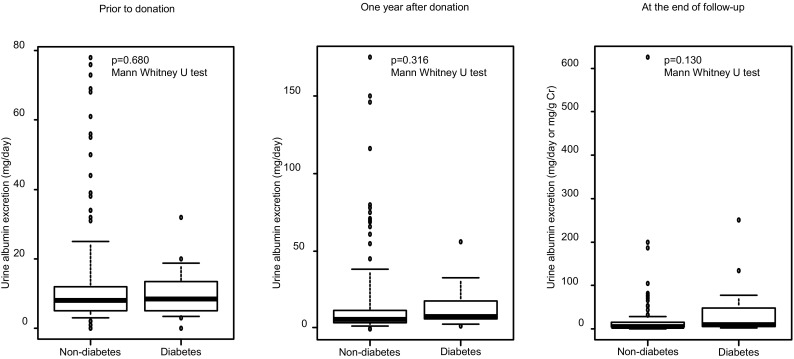
Urine albumin excretion prior to donation, 1 year after donation, and at the end of follow-up

**Fig. 3:**
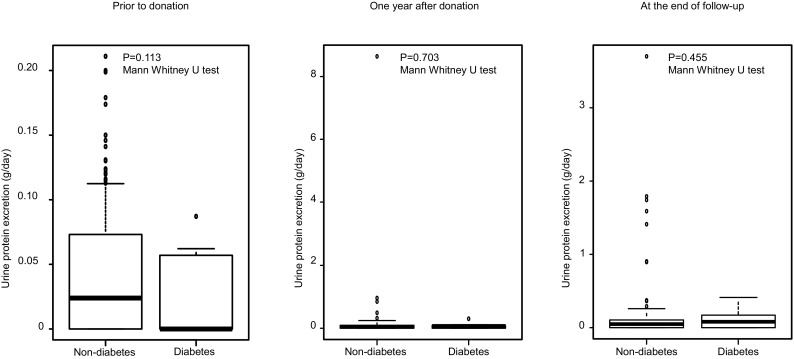
Urine protein excretion prior to donation, 1 year after donation, and at the end of follow-up

The median follow-up period was 4.3 (1.5–10.7) and 4.6 (1.0–13.0) years in donors with and without diabetes, respectively. EGFR and urine albumin and protein excretion at 1 year after donation and at the end of follow-up are shown in Figs. 1, 2, and 3. EGFR at 1 year after donation and at the end of follow-up was 52.0 ± 7.1 ml/min/1.73 m^2^ and 51.7 ± 7.1 ml/min/1.73 m^2^ in the diabetic group and 51.7 ± 10.9 ml/min/1.73 m^2^ and 52.1 ± 12.2 ml/min/1.73 m^2^ in the non-diabetic group, respectively. Urine albumin excretion at 1 year after donation and at the end of follow-up was 8 (2–56) and 9.5 (2–251) mg/day (or mg/g Cr) in the diabetic group and 6 (0–175) and 6 (0–626) mg/day (or mg/g Cr) in the non-diabetic group, respectively. Urine protein excretion at 1 year after donation and at the end of follow-up was 0.077 (0–0.30) and 0.079 (0–0.41) g/day in the diabetic group and 0.066 (0–8.6) and 0.051 (0–3.7) g/day in the non-diabetic group, respectively. There were no significant differences in these parameters between the diabetic group and non-diabetic group. In the non-diabetic group, there was a donor who had developed multiple myeloma and exhibited a urine protein excretion rate of 6.4 g/day at 1 year after donation and 3.7 g/day at the end of follow-up.

In the diabetic group, there were no data of 24-h urine albumin level at the end of the follow-up period in three cases, while one case had data for spot urine albumin. There were no data for 24-h urine protein levels at the end of follow-up in two cases. In the non-diabetic group, there were no data for eGFR at 1 year after donation in five cases, urine albumin excretion at 1 year after donation in 79 cases, and at the end of follow-up in 85 cases (there were data of spot urine albumin excretion in seven of the 85 cases), and urine protein excretion at 1 year after donation in 57 cases, and at the end of follow-up in 47 cases.

### Multivariate analysis

Table 4 shows multivariate analyses for parameters of pre-donation associated with low eGFR and high urine albumin excretion at the end of follow-up. Age ≥ 60 years and eGFR < 80 ml/min/1.73 m^2^ were associated with low eGFR at the end of follow-up. Only urine albumin excretion > 30 mg/day was associated with high urine albumin excretion at the end of follow-up.

**Table 4:** Logistic regression analysis for predicting low eGFR and high urine albumin excretion

Variable	Univariate analysis	Multivariate analysis		
	*p* value	Odds ratio	95% CI	*p* value
eGFR < 45 ml/min/1.73 m^2^				
Age (≥ 60/< 60)	0.00104	2.38	1.20–4.69	0.0127
Sex (female/male)	0.448			
BMI (≥ 25/< 25)	0.643			
Diabetes (yes/no)	0.359			
Hypertension (yes/no)	1.00			
Dyslipidemia (yes/no)	1.00			
Smoking (yes/no)	1.00			
eGFR (< 80/≥ 80)	< 0.001	10.9	5.20–23.0	< 0.001
Urine albumin excretion (≥ 30/< 30)	0.787			
Urine albumin excretion > 30 mg/day				
Age (≥ 60/< 60)	0.617			
Sex (female/male)	0.289			
BMI (≥ 25/< 25)	0.588			
Diabetes (yes/no)	0.153			
Hypertension (yes/no)	0.128			
Dyslipidemia (yes/no)	0.406			
Smoking (yes/no)	1.00			
eGFR (< 80/≥ 80)	0.200			
Urine albumin excretion (≥ 30/< 30)	< 0.001	108	11.9–993	< 0.001

*BMI* body mass index,* eGFR *estimated glomerular filtration rate

## Discussion

In this study, there were no significant differences in eGFR as well as urine albumin and protein excretion after kidney donation between the diabetic and non-diabetic groups. To the best of our knowledge, this is the first report of renal prognosis in living kidney donors with diabetes.

There have been contradictory reports that diabetes is a risk factor for the deterioration of renal function after nephrectomy for renal cell carcinoma or other illness [2–9]. However, these reports include cases of partial nephrectomy; cases with microalbuminuria, macroalbuminuria, and overt proteinuria; cases in which the stage of diabetic nephropathy is not clear; or cases in which there is no indication of the presence of proteinuria or albuminuria. Therefore, these reports are useful for reference, but cannot be applied to living kidney donors.

In The United Kingdom Prospective Diabetes Study (UKPDS 64), 7.3% of patients had microalbuminuria or worse at the time of diabetes diagnosis. This number increased to 17.3 and 24.9% after 5 and 10 years, respectively. Meanwhile, 0.7% of patients had overt proteinuria, which increased to 3.1 or 5.3% after 5 and 10 years, respectively [13]. In the current study, one of 14 donors with diabetes (7.1%) had microalbuminuria before donation and three of 12 donors (25%) had microalbuminuria at 4.3 (1.5–10.7) years after donation. This suggests that microalbuminuria is more likely to occur in donors with diabetes compared with general patients with diabetes. This may be due to glomerular hyperfiltration, which develops after loss of a kidney [14, 15]. One of the donors with diabetes who had developed microalbuminuria after donation was also the only case that also had hypertension, dyslipidemia, and a history of smoking among 14 donors with diabetes. Moreover, the donor was mildly obese. This indicated that living kidney donor candidates with diabetes who have microalbuminuria or multiple risk factors would not be suitable for kidney donation. However, another case in which microalbuminuria developed after donation was elderly (61 years of age) and did not have any other notable risk factors other than diabetes. Indeed, such case can develop microalbuminuria; hence, a careful follow-up is necessary.

Before donation, there was only one donor with microalbuminuria in the diabetic group. However, there were 17 donors with microalbuminuria in the non-diabetic group. The donors with microalbuminuria had a urine albumin excretion of 32 and 50 (31–78) mg/day in the diabetic group and in the non-diabetic group, respectively (*p* = 0.21). Although there were no significant differences, the urine albumin excretion was higher in the non-diabetic group. We considered that this was the reason microalbuminuria (at 1 year after donation and at the end of follow-up) seemed dominant in donors without diabetes (Fig. 2), and this was supported by the results of the multivariate analysis for albuminuria after donation (Table 4). Nevertheless, there were no significant differences in the percentages of microalbuminuria between the groups [at 1 year after donation, 18.2 and 12.9% in the diabetic group and in the non-diabetic group, respectively (*p* = 0.972); and at the end of follow-up, 25.0 and 10.6% in the diabetic group and in the non-diabetic group, respectively (*p* = 0.331)]. In the donors with microalbuminuria only, there were also no significant differences in the urine albumin excretion between the groups [at 1 year after donation, 44.5 (33–56) and 69.0 (31–175) mg/day in the diabetic group and in the non-diabetic group, respectively (*p* = 0.319) and at the end of follow-up, 134.0 (77–251) and 70.5 (32–626) mg/day in the diabetic group and in the non-diabetic group, respectively (*p* = 0.186)].

This study has some limitations. First, the sample size of donors with diabetes was not large. Second, follow-up periods of 4.3 (1.5–10.7) years in the diabetic group might be too short to observe the course of diabetic nephropathy. Therefore, further observation over a longer period is needed. Third, there were many cases in which data of urine albumin or protein excretion were not included.

## Conclusions

In this study, there were no significant differences in eGFR as well as urine albumin and protein excretion after kidney donation between donors with and without diabetes. Individuals with diabetes who have multiple risk factors of chronic kidney disease or microalbuminuria should not donate a kidney.
